# Initial Effects of the National PCV7 Childhood Immunization Program on Adult Invasive Pneumococcal Disease in Israel

**DOI:** 10.1371/journal.pone.0088406

**Published:** 2014-02-07

**Authors:** Gili Regev-Yochay, Galia Rahav, Klaris Riesenberg, Yonit Wiener-Well, Jacob Strahilevitz, Michal Stein, Daniel Glikman, Gabriel Weber, Israel Potasman, Ron Dagan, for the IAIPD study group

**Affiliations:** 1 Infectious Disease Unit, Sheba Medical Center, Ramat-Gan, Israel; 2 Infectious Disease Soroka University Medical Center, Beer-Sheva, Israel; 3 Infectious Disease Unit, Shaare Zedek Medical Center, Jerusalem, Israel; 4 Department of Clinical Microbiology & Infectious Disease, Hadassah-Hebrew University, Jerusalem, Israel; 5 Pediatric Infectious Disease Unit, Wolfson Medical Center, Holon, Israel; 6 Pediatric Infectious Disease Service, Western Galilee Hospital, Nahariya, Israel; 7 Infectious Disease Unit, Carmel Medical Center, Haifa, Israel; 8 Infectious Disease Unit, Bnei-Zion Medical Center, Haifa, Israel; 9 Pediatric Infectious Diseases Unit, Soroka University Medical Center & Faculty of Health Sciences, Ben-Gurion University of the Negev, Beer-Sheva, Israel; Food and Drug Administration, United States of America

## Abstract

**Background:**

PCV7 was introduced as universal childhood vaccination in Israel in July 2009 and PCV13 in November 2010. Here we report data on adult invasive pneumococcal disease (IPD), two years post PCV7 implementation and before an expected effect of PCV13.

**Methods:**

An ongoing nationwide active-surveillance (all 27 laboratories performing blood cultures in Israel), providing all blood & CSF *S. pneumoniae* isolates from persons >18 y was initiated in July 2009. Capture-recapture method assured reporting of >95% cases. All isolates were serotyped in one central laboratory. IPD outcome and medical history were recorded in 90%. Second year post PCV implementation is compared to the first year.

**Results:**

During July 2009 to June 2011, 970 IPD cases were reported (annual incidence [/100,000] of 9.17 and 10.16 in the two consecutive years, respectively). Respective case fatality rates (CFRs) were 20% and 19.1%. Incidence of IPD and CFR increased with age and number of comorbidities. Incidence rate was significantly greater during the second winter, 7.79/100,000 vs. 6.14/100,000 in first winter, p = 0.004, with a non-significant decrease during summer months (3.02 to 2.48/100,000). The proportion of IPD cases due to PCV7-serotypes decreased from 27.5% to 13.1% (first to second year) (p<0.001). Yet, non-PCV13-strains increased from 32.7% to 40.2% (p = 0.017). The increase in non-PCV13-strains was highly significant in immunocompromised patients and to a lesser degree in non-immunocompromised at risk or in older patients (>64 y). Among younger/healthier patients serotype 5 was the major increasing serotype. Penicillin and ceftriaxone resistance decreased significantly in the second year.

**Conclusions:**

While overall annual incidence of IPD did not change, the indirect effect of PCV7 vaccination was evident by the significant decrease in PCV7 serotypes across all age groups. Increase in non-VT13 strains was significant in immunocompromised patients. A longer follow-up is required to appreciate the full effect of infant vaccination on annual IPD.

## Introduction

Pneumococcal infections, including invasive pneumococcal diseases (IPD), such as bacteremia and meningitis, cause a significant morbidity and mortality in adults [1]. Until recently, the only direct prevention measure available was vaccination with the 23-valent polysaccharide vaccine (PPSV23) of older adults (>60 y) and younger adults with risk factors for severe IPD. Yet, the efficacy and effectiveness of PPSV23 in these populations are controversial [2], [3].

In 2000, a 7-valent pneumococcal conjugate vaccine (PCV7) was introduced to the national pediatric immunization program (NIP) in the USA. This resulted in a rapid and dramatic decrease in IPD, both in children and in adults (through an indirect, herd protection effect) [4]. These indirect effects in Europe were not always consistent with the USA experience and varied from country to country [5]–[9]. In some countries increases in non-vaccine type (NVT) strains causing infection largely offset the reduction in PCV7 vaccine serotypes (VT7) [5], [7], [10]–[12].

The recent approval of PCV13 for adult populations has raised a new controversy. While many anticipate that PCV13 introduction to adults will play a major role in IPD prevention, others suggest that the indirect effect of vaccinating children will be significant enough to eliminate the need for adult vaccination, and others speculate that replacement of VT7 strains by non VT7 (NVT7) strains may reduce the significance of the vaccine effect [4], [5], [13], [14].

Another unresolved issue is, whether different patterns of serotype distribution that will follow vaccine implementation will result in changes in disease characteristics (such as increase in empyema incidence), as some have suggested [12], [15]–[17].

In Israel, PCV7 was implemented in the NIP on July 2009 (with a 2, 4 and 12 month infant schedule, and a 2-dose catch-up plan in the second year of life). It was gradually replaced by PCV13 since November 2010, with no catch-up plan for PCV13. Pneumococcal polysaccharide vaccine (PPSV23) is recommended in Israel to adults with IPD predisposing comorbidities (i.e., diabetes mellitus, chronic renal failure, congestive heart failure, lung disease, HIV, immunodeficiency, asplenic state, hematogenic malignancy and prior neurosurgery) and to all adults >65 y. Uptake of PPSV23 among this population is ∼70% (Maccabi Healthcare Services HMO data).

To assess the indirect effects of the introduction of PCV7 childhood immunization on adult IPD, we conducted a nationwide surveillance study of adult IPD in Israel. Here we report the results of the first two years following PCV7 implementation, before any expected effect of PCV13.

## Methods

### Ethics Statement

The study was conducted following protocols approved by the Sheba Medical Center Institutional Review Board (IRB) and the Soroka University Medical Center IRB. Since this was a retrospective observational study, the institutional review board waived the need for written informed consent from the participants and informed consent was not obtained. Therefore, all patient records/information was anonymized and de-identified prior to analysis.

### Study Period

An ongoing nationwide, prospective, population-based, active surveillance was initiated on July 1, 2009. Results from the first two years (until June 30, 2011) are reported here.

### Study Population

In 2009 and 2010, the Israeli adult population (>18 years old) consisted of 5,029,600 and 5,119,200 individuals respectively. The surveillance included all 27 laboratories and medical centers that routinely obtain blood and cerebrospinal fluid (CSF) cultures: All 26 hospitals and one major outpatient health maintenance organization (HMO); Maccabi Healthcare Services, central laboratory. Less than 1% of blood cultures and no CSF cultures are obtained outside these centers. This enabled us to cover practically all culture-confirmed IPD cases in the Israeli adult population.

### Case Definition

An IPD case was defined by isolation of *S. pneumoniae* from blood or CSF. Diagnoses based solely on non-culture methods (polymerase chain reaction, antigen testing, gram stain or clinical diagnosis only) were excluded. Positive cultures from sterile sites other than blood or CSF (i.e. joint/pleural fluid/peritoneal fluid) were also excluded [18].

### Evaluation of Vaccine Uptake

Estimates of PCV7 coverage before NIP implementation were based on sale figures provided by the distributer. The methodology of evaluating vaccine uptake initiated in July 2009 was described elsewhere [18].

### Isolate Collection

In order to assure >95% reporting, several collecting methods were carried out; all invasive *S. pneumoniae* isolates are required to be reported and sent to the Ministry of Health (MOH) reference laboratory by law. In addition to this passive surveillance an active surveillance using a capture-recapture method took place as described previously [18].

### Data Collection

Data from the hospital medical files of all cases were collected retrospectively within 2–3 month of hospitalization by investigators of the IAIPD group. Data collected included: socio-demographic data (gender, age, place of birth, city of residence), medical history including: any comorbidity, IPD predisposing comorbidities (diabetes mellitus, chronic renal failure, congestive heart failure, lung disease, HIV, immunodeficiency, asplenic state, malignancy and prior neurosurgery), substance abuse, smoking history, influenza in the preceding days before hospitalization and vaccination history. Data on antibiotic treatments, in-hospital complications (septic shock, need for ventilation, disability as determined by transition to a long term care facility and mortality) were also collected. Influenza like illness (ILI) surveillance data, were retrieved from the Israeli Center for Disease Control (ICDC) registry (http://www.old.health.gov.il).

### Laboratory Examinations

Susceptibility testing of the isolates was performed at the local laboratory of each medical center. All centers assessed susceptibility to penicillin, ceftriaxone and erythromycin following Clinical and Laboratory Standards Institute guidelines (CLSI) (http://www.clsi.org/source/custom/currentdocs.cfm) most centers assessed susceptibilities to a fluoroquinolones, different centers assessed different respiratory fluoroquinolones. Serotyping was performed to all viable isolates, received by the headquarter laboratory at the Pediatric Infectious Disease laboratory at the Soroka University Medical Center using the Quellung reaction (Staten Serum Institute, Copenhagen, Denmark).

### Statistical Analysis

Incidence and mortality rates were calculated using the data of the Israeli Central Bureau of Statistics. Case fatality rates were calculated as the percent of mortality cases of IPD cases. To define predictors for mortality and factors associated with NVT13 disease, multivariate logistic models were used. Potential predictors for mortality were: age, sex, predisposing comorbidities, non-hematologic metastatic malignancies, smoking and the source of infection. Predictor variables that were significantly associated with mortality (p<0.2) in a univariate analysis were included in the model. Adjusted odds ratios (aORs), 95% confidence intervals (CIs) and adjusted p-values were calculated. SAS 9.2 software was used.

## Results

From July 2009 (at the time of introduction of PCV7 into the national immunization program), until July 2011, 970 IPD cases were reported in adults; 460 during 2009–10 and 520 during 2010–11. In June 2009 and June 2010, vaccine uptake of ≥2 PCV doses was 20%, and 71% respectively, and by June 2011 96% of children 12–23 months old received ≥2 PCV doses. Uptake of ≥3 doses for children 18–23 months old was 10%, 25% and 83% respectively.

### Incidence and CFR

**Figure 1:**
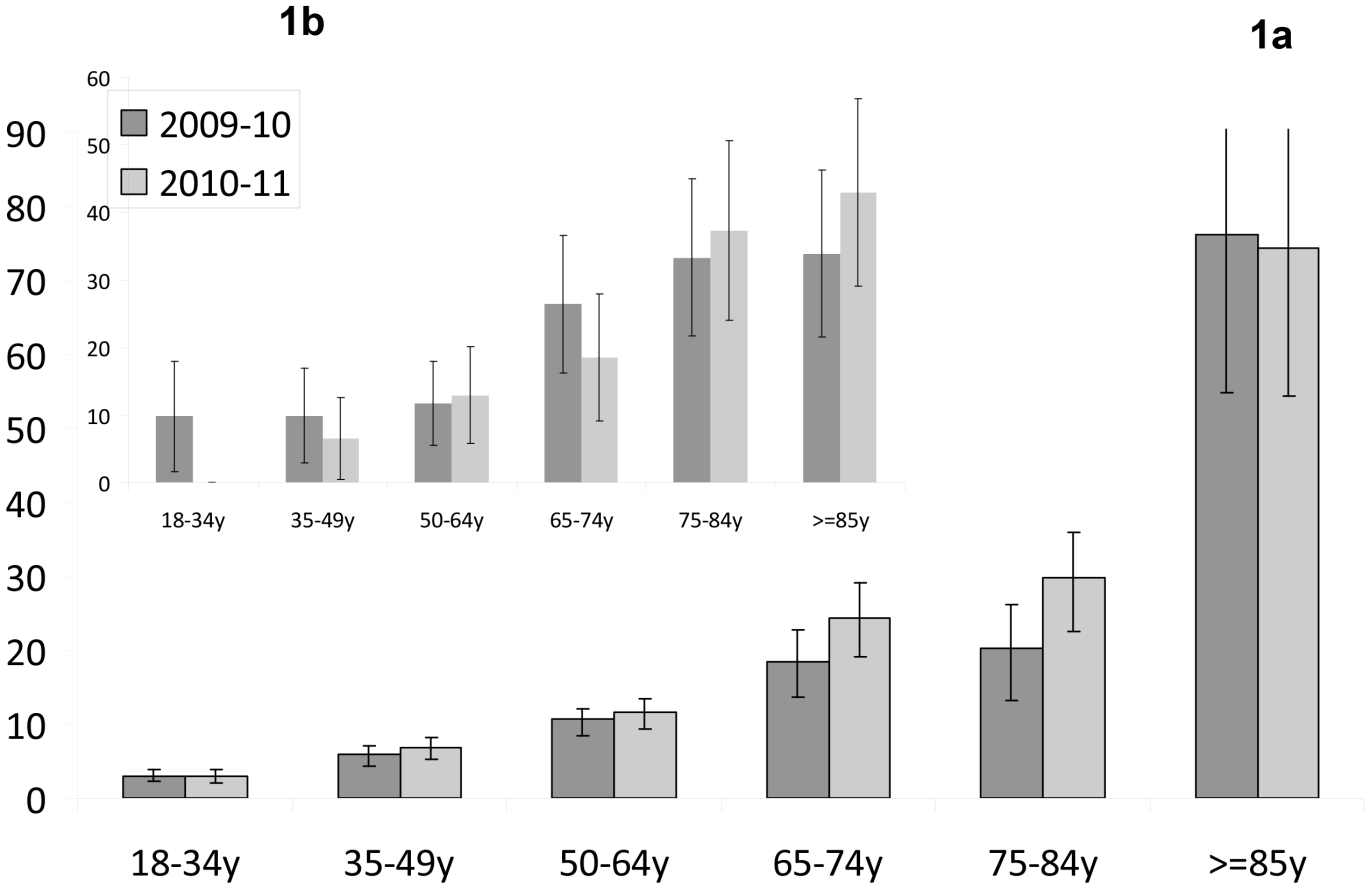
Adult IPD cases in Israel by age group. a. Incidence rate and b. Case fatality rate. Black bars – First study year (2009–10), light grey – Second study year (2010–11).

The annual incidence of IPD per 100,000 was 9.15 during the first year and 10.16 during the second year (**Figure 1a** **).** The respective case fatality rates (CFRs) were 20% and 19.1%. The incidence increased with age (**Figure 1a**) and with the number of comorbidities (data not shown), with no difference between the first and second study years. The incidence of overall IPD did not decrease in any of the age groups during the second year. CFR increased with age (**Figure 1b**) and comorbidies (data not shown). During the second year there were significantly less cases of bacteremia with an unrecognized source of infection (14% VS. 20%, P = 0.026, 2^nd^ vs. 1^st^ y) and slightly more single lobe pneumonias (54% vs 48%, p = 0.07), the incidence of empyema cases did not change (**Table S1**). CFR was not correlated with month or season and the overall CFR did not change between the two years. Yet, CFR among the younger age group (<50 y) decreased significantly from 10.7% in the first year to 3.1% in the second year (p = 0.04) (**Figure 1b**).

### Change in Serotype Distribution

**Figure 2:**
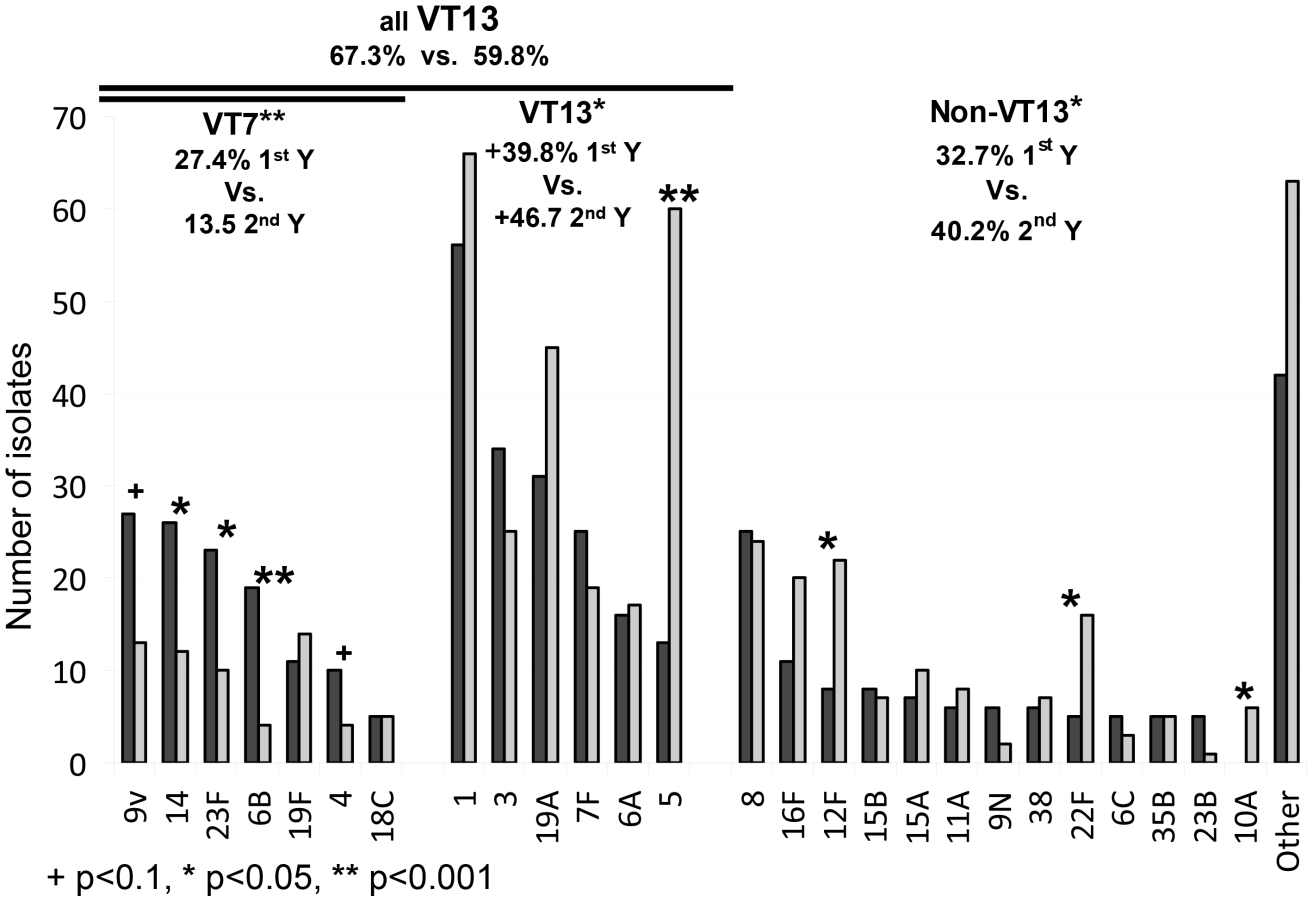
Serotype distribution of IPD cases in the two consecutive study years. Dark grey – First year (2009–10), light grey – Second year (2010–11).

While incidence of all IPD cases did not change, incidence of VT7 IPD decreased significantly from 27.5% in the first year to 13.1% (p<0.001) in the second year **(** **Figure 2**). This decrease was accompanied by an increase in NVT7 strains. Serotypes not included in PCV7 but included in PCV13 (addVT13) increased from 39.8% to 46.7% (p = .033), Serotypes not included in PCV13 (NVT13) increased from 32.7% to 40.2% (p = 0.017). The most striking increase was observed in serotype 5 (included in PCV13 but not PCV7); from 13 annual cases, i.e. 0.26/100,000 and 2.8% of all isolates in the first year to 60 annual cases, i.e., 1.17/100,000 and 11.5% of all isolates in the second year (P<0.001). The increase in serotype 5 IPD began in December 2010 and reached its peak in March 2011, when it consisted 18.5% of all isolates in that month. It is yet to be seen whether this outbreak has been fully resolved. The outbreak of serotype 5 was not localized to a specific hospital or region, but was observed initially in hospitals from Southern and Central Israel and later spread to the Northern region of Israel. NVT13 strains that increased significantly during the second year include 10A, 22F and 12F. None of the NVT13 strains significantly decreased.

**Figure 3:**
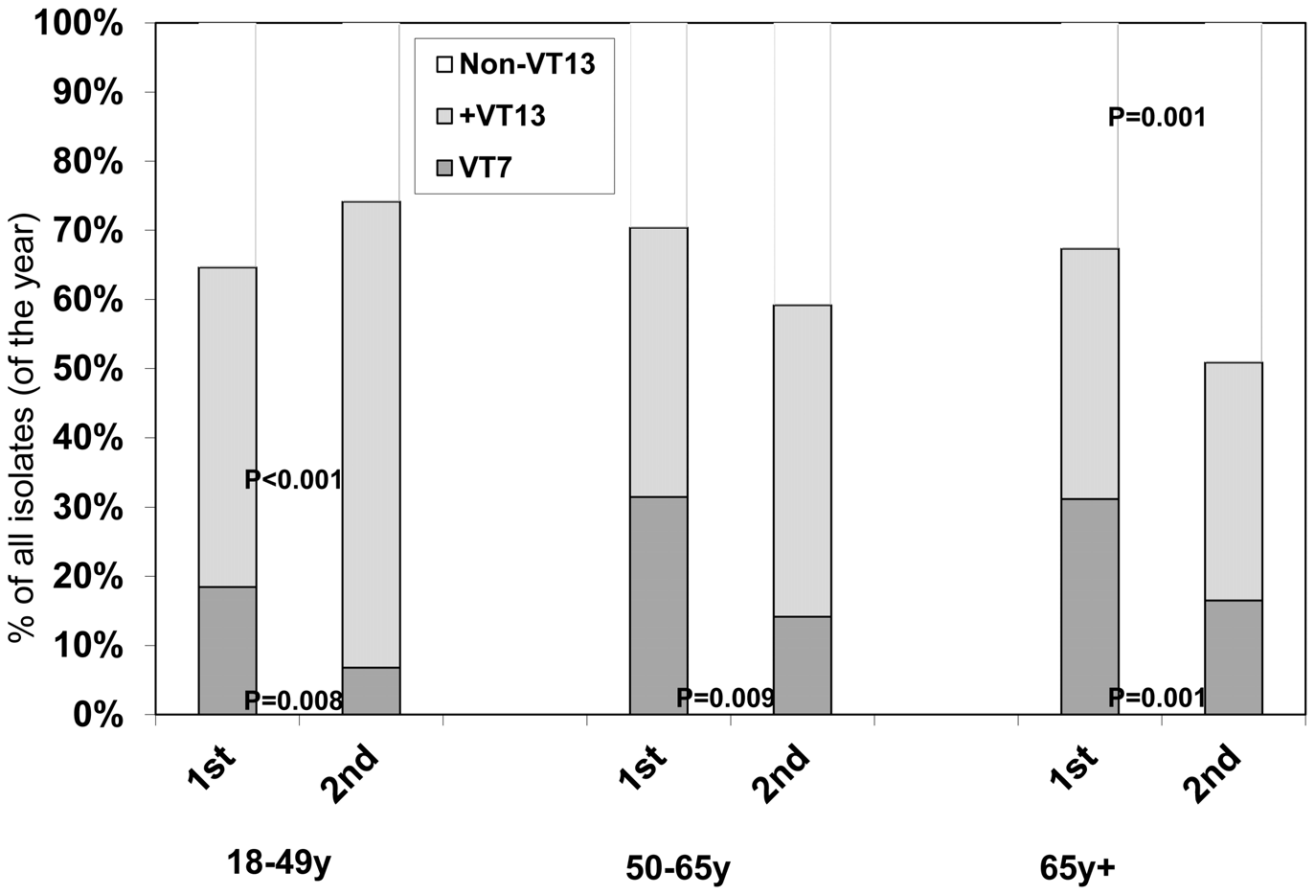
IPD cases by age and vaccine serotype coverage. Dark grey – serotypes included in PCV7 (VT7), light grey – serotypes included in PCV13 (but not 7) (+VT13), white – serotypes not included in either PCV7 or PCV13 (Non-VT13).

The indirect effect of PCV7 administration to infants was evident by a significant reduction of VT7 IPD cases in adults. This was significant across all age groups and health status. Yet, while in younger (18–50 years) and healthier adults the decrease in VT7 cases was mainly accompanied by an increase in serotypes included in PCV13, particularly serotype 5; in the elderly or chronically ill patients (defined by having a comorbidity for which PPSV23 is recommended), the VT7 decrease was mainly accompanied by an increase in non-VT13 strains (**Figure 3a and 3b**). In a multivariate regression, the significant variables associated with NVT13 cases were second year of the study (OR:1.369, 95%CI 1.03–1.83) and immunocompromised patients (OR:1.963, 95%CI 1.34–2.87); while older age (>65 years) only trended to significance (OR: 1.34, 95%CI 0.94–1.91), as was having comorbidities other than being immunocompromised (smoker, IVDU, lung disease) (OR1.39, 95%CI 0.95–2.02). Other variables included in the model were gender and seasonality.

### Seasonality

**Figure 4:**
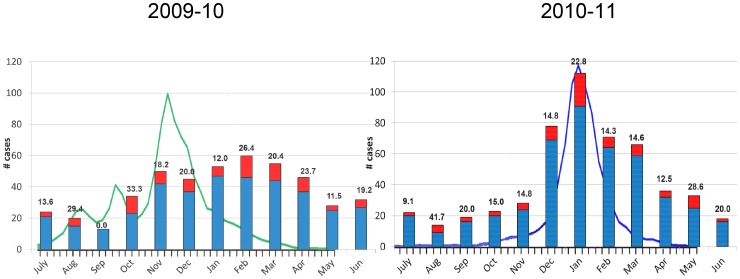
Seasonality effect; monthly IPD cases (bars) and weekly ILI cases (line). Blue: IPD cases that were discharged from the hospital, Red: In hospital mortality cases.

As expected, a seasonal variation in IPD incidence was observed, with a peak incidence each winter. To assess whether seasonality of IPD was related to the influenza season, IPD monthly incidence was plotted against the weekly influenza like illness (ILI) report obtained by the Israeli CDC (**Figure 4**). During the first study year the 2009 nH1N1 pandemic occurred with a particularly prolonged influenza season and higher morbidity and mortality of younger patients. During the second year a shorter but more severe influenza season was observed, with higher ILI incidence in a shorter period, the predominant influenza that year was still A/nH1N1, but with co-occurrence of A/H3N2 as well as high incidence of Respiratory Syncytial Virus (RSV) concomitantly. IPD incidence rate correlated, with some delay, with weekly ILI reports (**Figure 4**). IPD incidence was significantly greater in the second winter, 7.63/100,000 vs. 6.14/100,000 in first winter, p = 0.004, with a non-significant decrease in summer months (3.00 to 2.52/100,000).

### Early Effects on Antibiotic Resistance

**Figure 5:**
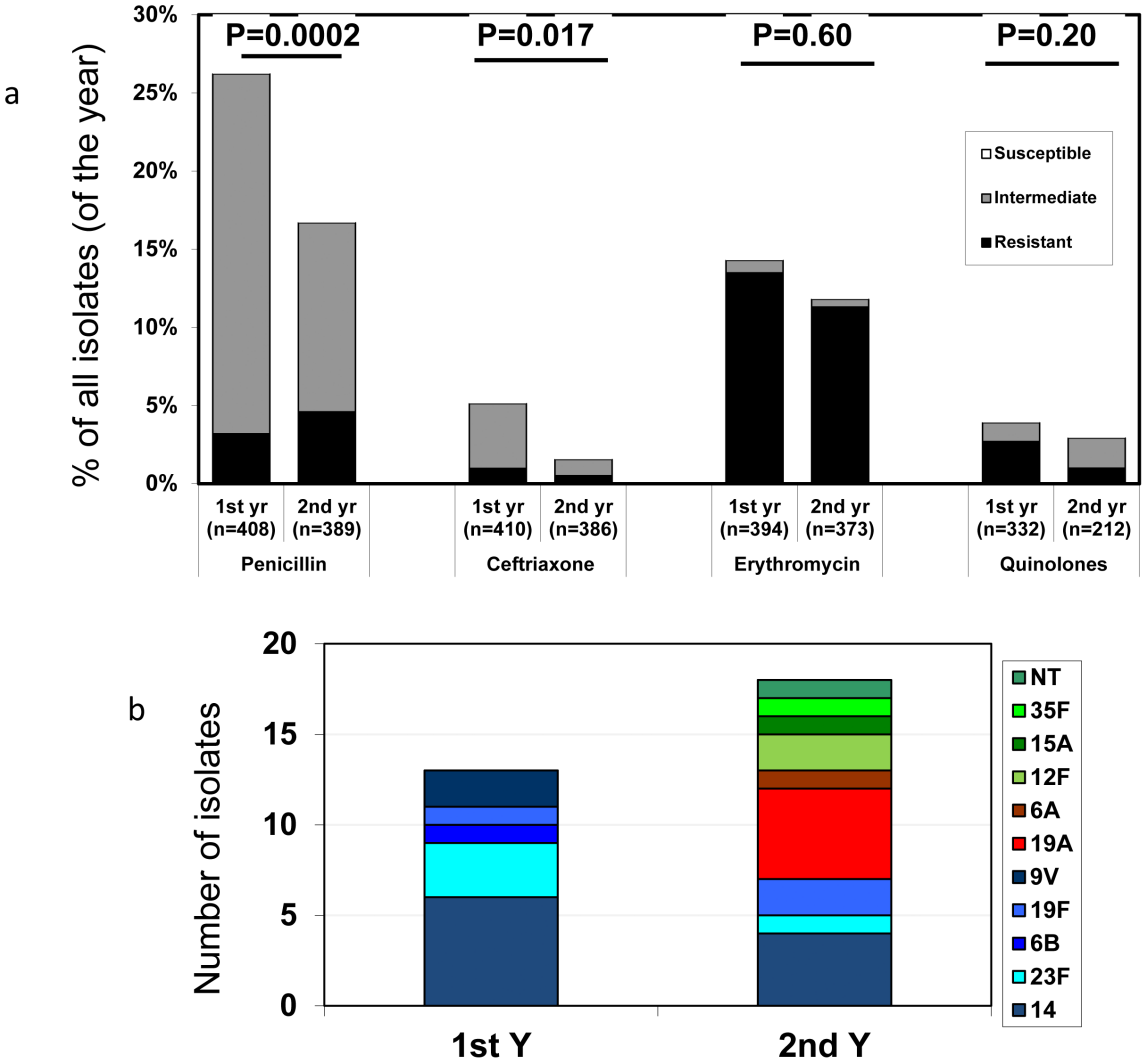
Antibiotic susceptibility of IPD strains. a. Antibiotic susceptibility to different antibiotic classes. Black – resistant, Grey – intermediate, White – susceptible. b. Serotype distribution of isolates with penicillin MIC≥2 µg/ml, Blue shades- VT7 strains, Red shades – strains covered by PCV13, but not PCV7 (+VT13). Green shades – NonVT13 strains.

During the second year, a significant decrease in proportion of isolates with penicillin MIC ≥0.125 µg/ml and isolates resistant to ceftriaxone was observed (from 26.2% to 16.4% and from 5% to ∼1%, respectively. Yet, the number of isolates with penicillin MIC ≥2 µg/ml tended to increase (from 13 isolates (3.2%) to 21 (4.7%), P = 0.27) (**Figure 5a**). As previously reported [19], during the first study year, most isolates with penicillin MIC ≥0.125 µg/ml and all isolates with MIC ≥2 µg/ml, belonged to VT7 serotypes. Indeed, VT7 resistant strains decreased, however, this decrease was offset by an increase in NVT7 strains with penicillin MIC ≥2 µg/ml; most striking was the increase in serotype 19A (**Figure 5b**).

## Discussion

The indirect overall effect of introducing PCV to the national pediatric immunization program on adult IPD incidence has been debatable. While reports from the USA showed rapid and immense impact [4], reports from Europe varied [5], [7], [8], [10]. Here, we report the initial effects of PCV7 pediatric vaccination on adult IPD incidence and mortality in a national active surveillance study.

Two years after introduction of PCV7 to the NIP in Israel, IPD incidence in adults has not changed. However, a significant effect of PCV7 was observed. VT7 strains decreased significantly, in all age groups and regardless of comorbidities or health status. However, increase in NVT7 strains was observed: among younger and healthier adults the increase was mainly by the epidemic serotype 5. However, among older and chronically ill adults, with comorbidities for which PPSV23 is indicated, the increase was by strains belonging to NVT13.

Serotype 5 is an epidemic serotype, with typical local epidemics lasting several months to a few years each [20], [21]. Thus, the expected decline in this serotype may result in a significant decrease in total IPD incidence among younger adults in Israel in the near future, in particular since it is also contained in the PCV13 formulation.

In the USA and Europe, the indirect effect of childhood PCV7 vaccination on IPD among adults ≥65 y was gradual with only a relatively mild decline in VT7 IPD during the first year post-PCV7 implementation. Here, we observed a 50% decrease in VT7 IPD cases within the first year of PCV7 implementation. However, the observation that the effect of PCV7 in the elderly and in chronically ill adults is offset by the increase in NVT13 strains is somewhat discouraging. A similar observation was recently reported by Liesenborghs et al. in Belgium [22]. The significant increase in NVT13 cases in the elderly, mainly in immunocompromised adults, may be explained by the fact that the relatively low virulent strains that occupy the nasopharynx niche in the post-PCV era and which rarely invade or cause infections in younger and healthier adults, do cause disease in the vulnerable group of elderly and chronically ill adults. In that case, only a vaccine that will cover all pneumococcal serotypes (like future protein pneumococcal vaccines) will decrease overall IPD-related morbidity in this population. Further studies and follow-up on the epidemiology of IPD in the elderly and chronically ill patients in the post-PCV7 era are required.

An interesting finding was the relative decrease in mortality among the younger patients during the second study year as compared to the first. This could be an effect of PCV7, due to a significant decrease in VT7 strains in this age group. However, the greater mortality rates of younger adults in the first study year, could be due to the high susceptibility of young adults to the pandemic A/nH1N1 influenza that occurred during that year [23], returning to baseline low mortality in the young during the second study year. Unfortunately, we do not have data from the years prior to PCV7 implementation that would determine whether that is the case. The fact that in the second year a rise in influenza virus A/H3N2 was observed compared to the first year may also explain the lack of decrease of IPD in the elderly since A/H3N2 has been associated with infection in older adults [24].

Influenza seasonality has been shown to influence IPD incidence mainly in adults [25], [26]. To correctly assess the real PCV effects that are unrelated to influenza seasonality we compared non-winter periods of the year. This comparison illustrated a slight, not yet statistically significant decrease in IPD incidence in the second year.

Another effect of PCV7 observed in our study, was the effect on prevalence of antibiotic resistance. A significant reduction in penicillin-nonsusceptible *S. pneumoniae* (PNSSP) was observed. This effect was due to the decrease in VT7 strains that initially constituted most of the antibiotic-resistant strains. This finding is not unexpected since similar findings have been reported from different geographic regions following PCV7 introduction [27]–[30].

In many regions where PCV was introduced, re-emergence of antibiotic resistant *S. pneumoniae* followed the initial decrease [31], [32]. This antibiotic resistance re-emergence was typically delayed and not immediate [33]. The increase in antibiotic resistance was shown to be mainly due to expansion of previously relatively distinct clones of antibiotic-resistant NVTs, particularly serotypes 19A, 15A, and 35B as well as increase in resistance within serotypes but less so due to capsular switching or de-novo acquisition of resistance genes [31], [33], [34]. In our study, the only VT7 resistant serotype that increased was 19F, and similar to other studies we showed an increase in MDR 19A. Yet, the disturbing observation was that several highly penicillin-resistant NVT13 (MIC ≥2.0 µg/ml) that were not detected in the first year have emerged in the 2^nd^ year. It is yet to be seen, whether this trend will become significant and whether these serotypes will further emerge in the coming years. This early emergence of antibiotic resistant serotypes further demonstrates that vaccination alone, without concomitant decrease in antibiotic use, will not result in long term reductions in antibiotic resistance among pneumococci.

Our study is limited in assessing only the first two years post-PCV7 introduction. To observe the full indirect effects of childhood PCV vaccination on adult IPD a longer term follow-up is required.

## Supporting Information

Table S1 **Site of Infection.**
(DOCX)
