# Supplementary material for: Initial Effects of the National PCV7 Childhood Immunization Program on Adult Invasive Pneumococcal Disease in Israel
Source: PLoS One. 2014 Feb 7;9(2):e88406. doi: 10.1371/journal.pone.0088406 (PMC3917916; doi:10.1371/journal.pone.0088406)
Supplement: Table S1 — Site of Infection. (DOCX) [file pone.0088406.s001.docx]

**Table S1: Site of infection*:**

|  | | |  | **1^st^ Year (n=416**)** | | **2^nd^ Year (n=468***)** | |
| --- | --- | --- | --- | --- | --- | --- | --- |
|  | | **# cases**  **(% of all IPD cases)** | | | **# mortality cases**  **(% mortality)** | **# cases**  **(% of all IPD cases)** | **# mortality cases**  **(% mortality)** |
| Pneumonia | | | | 199  (47.8%) | 29  (14.6%) | 252  (53.8%) | 35  (13.9%) |
|  | Single lobe | | |  |  |  |  |
|  | Bi-lobar | | | 102  (24.5%) | 29  (28.4%) | 99  (22.2%) | 23  (23.2%) |
|  | Empyema | | | 14  (3.4%) | 0  (0%) | 22  (4.7%) | 1  (4.5%) |
| All Lung infections | | | | 301  (72.4%) | 58  (19.3%) | 351  (75.0%) | 59  (16.8%) |
| Meningitis | | |  | 28  (6.4%) | 2  (7.1%) | 33  (7.1%) | 6  (18.2%) |
| Sinusitis | | |  | 3  (0.7%) | 0  (0%) | 3  0.7%) | 1  (33.3%) |
| Bacteremia with no source | | |  | 84  (20.2%) | 24  (28.6%) | 68  (14.5%)†† | 14  (20.6%) |
| Other † | | |  | 15  (3.6%) | 1  (6.7%) | 13  (2.8%) | 1  (7.7%) |

*not mutually exclusive, multiple infection sites were reported in some cases

**from 416 cases with diagnoses data

*** from 468 cases with diagnoses data

† Other includes: vascular abscesses, endocarditis, peritonitis, endometritis/pelvic inflammation, ear/nose/throat (ENT) infections, not otherwise diagnosed.

† †Significant change compared to 1^st^ year (p<0.05)
